# Structural and Functional Analysis of the Symmetrical Type I Restriction Endonuclease R.EcoR124I_NT_

**DOI:** 10.1371/journal.pone.0035263

**Published:** 2012-04-06

**Authors:** James E. N. Taylor, Anna Swiderska, Jean-Baptiste Artero, Philip Callow, Geoff Kneale

**Affiliations:** 1 Institute of Biomedical and Biomolecular Sciences, University of Portsmouth, Portsmouth, United Kingdom; 2 Partnership for Structural Biology, Institut Laue-Langevin, Grenoble, France; 3 Macromolecular Structure Research Group, Keele University, Keele, Staffordshire, United Kingdom; New England Biolabs, Inc., United States of America

## Abstract

Type I restriction-modification (RM) systems are comprised of two multi-subunit enzymes, the methyltransferase (∼160 kDa), responsible for methylation of DNA, and the restriction endonuclease (∼400 kDa), responsible for DNA cleavage. Both enzymes share a number of subunits. An engineered RM system, EcoR124I_NT_, based on the N-terminal domain of the specificity subunit of EcoR124I was constructed that recognises the symmetrical sequence GAAN_7_TTC and is active as a methyltransferase. Here, we investigate the restriction endonuclease activity of R. EcoR124I_NT_ *in vitro* and the subunit assembly of the multi-subunit enzyme. Finally, using small-angle neutron scattering and selective deuteration, we present a low-resolution structural model of the endonuclease and locate the motor subunits within the multi-subunit enzyme. We show that the covalent linkage between the two target recognition domains of the specificity subunit is not required for subunit assembly or enzyme activity, and discuss the implications for the evolution of Type I enzymes.

## Introduction

Restriction–modification (RM) enzymes act as a bacterial defence mechanism against foreign DNA. Host DNA is fully methylated at specific sequences by a methyltransferase (MTase), thus protecting its DNA from restriction by the accompanying endonuclease (ENase). Foreign DNA is cleaved by the ENase since it is unmethylated at these sites [1], [2].

Type I RM systems play a role in modulating horizontal gene transfer and thus are important in the spread of antibiotic resistance genes in bacterial populations [3]. They comprise large multi-subunit enzyme complexes encoded by three hsd (host specificity of DNA) genes, corresponding to three polypeptides: HsdS, responsible for DNA recognition, HsdM for DNA modification and HsdR for cleavage [3]. The ENase requires all three subunits (referred to hereafter as M, S and R) while the MTase requires just the M and S subunits, the stoichiometry being R_2_M_2_S and M_2_S, respectively.

For enzyme activity, the MTase is dependent upon S-adenosylmethionine, while the ENase in addition requires Mg^2+^ and ATP. The endonuclease acts as a molecular motor, translocating up to 50,000 bp of DNA. The translocation is driven by the hydrolysis of ATP, and leads to the extrusion of large DNA loops prior to cleavage. Both the nuclease and the ATPase domains are located in the R (or “motor”) subunits of the ENase. Crystal structures exist for a number of S, M and R subunits from various Type I systems [4]–[8]. However, no crystal structures have yet been reported for either a Type I MTase or an ENase, although a number of low-resolution models, supplemented by molecular modelling, have been reported [9]–[12].

Unlike typical Type II restriction enzymes that commonly have symmetric (palindromic) recognition sites, Type I enzymes recognise asymmetrical DNA sequences. The EcoR124I Type I RM system recognises the asymmetric DNA sequence GAAN_6_RTCG and both the pentameric ENase (R.EcoR124I) and the trimeric MTase (M.EcoR124I) have been well characterised [13], [14]. The GAA and RTCG sequences are recognised by the N-terminal and C-terminal target recognition domains (TRDs) respectively, and the length of the DNA spacer is determined by the length of the conserved coiled-coil region that separates the TRDs. However, structural analysis of the full-length S subunit of EcoR124I has been hampered by the insolubility of this subunit unless co-expressed with the M subunit [15]–[16].

A number of fragments of the HsdS gene have been generated by PCR and over-expressed [17]. A soluble subunit, denoted S_NT_, spanning residues 1–215 of the parent S subunit, contains the N-terminal TRD and the central conserved region [17] and has been shown to interact with the M subunit to form a functional MTase. This domain recognises the GAA of the parent recognition sequence and dimerises to recognise the symmetrical sequence GAAN_7_TTC [18]. This engineered RM system containing the N-terminal half of the S subunit was denoted by the subscript NT. We have previously shown that M.EcoR124I_NT_ has methylation activity *in vitro*, which can be inhibited by the Ocr (overcome classical restriction) protein. Through the use of small-angle neutron scattering, we constructed a model showing the location of the M and S subunits within the DNA methyltransferase [11].

In this paper, we report the restriction endonuclease activity of this symmetrical Type I enzyme, R.EcoR124I_NT_. We have characterised the cleavage of both linear and supercoiled plasmids containing one and two recognition sites, and confirmed the subunit assembly from the MTase “core”, via a R1 intermediate, through to the full 405 kDa R2 complex. Finally, we have used small-angle neutron scattering, utilising selective deuteration and contrast variation methods to obtain the low-resolution structure of the restriction endonuclease R.EcoR124I_NT_.

## Results

### Restriction Endonuclease Activity

**Figure 1:**
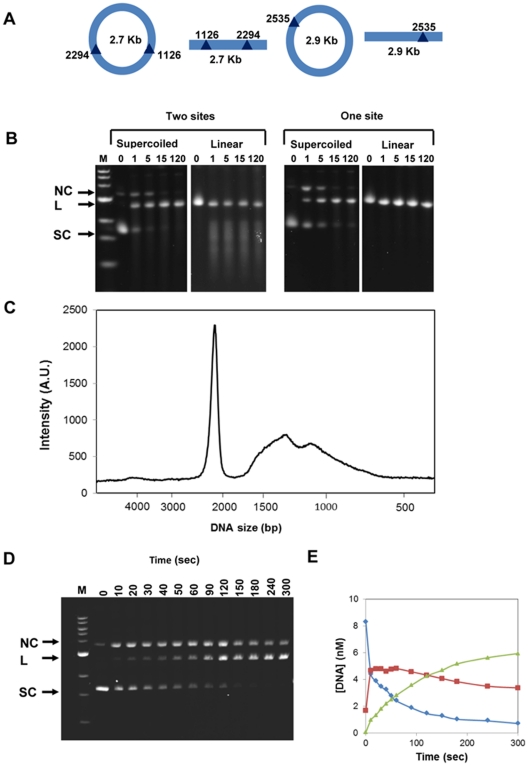
DNA cleavage of supercoiled and linear DNA with one or two R.EcoR124I_NT_ recognition sites. **(a)** Diagrammatic representation of the DNA substrates used: pUC19 (left) and pTK-neo (right). **(b)** Restriction assay. The endonuclease was incubated with DNA substrates containing either one or two recognition sites, both supercoiled and linear. Reactions were carried at 37°C and stopped with the addition of 0.5 M EDTA at 1, 5, 15 and 120 minutes. Reactions were run on a 0.8% TBE agarose gel with a 1kb DNA marker (NEB). **(c)** Densitometry scan of the reaction product of R.EcoR124I_NT_ incubated with two-site linear DNA. **(d)** Kinetics of cleavage of supercoiled DNA with two recognition sites (pUC19). Reaction products were analysed on a 0.8% TBE agarose gel. Reactions were carried at 37°C and stopped with the addition of 0.5 M EDTA at the time points shown over the range 10–300 secs. M represents a 1kb DNA marker (NEB). **(e)** Quantitation of supercoiled DNA (blue), nicked circle (red) and linear DNA (green) over the time-course of the reaction, by analysis of the data shown in (d).

The restriction endonuclease activity of R.EcoR124I_NT_ was investigated on different substrates and under a variety of conditions (Fig. 1). The wild-type restriction endonuclease requires a number of cofactors: ATP, MgCl_2_, and possibly AdoMet [19]. The requirement for these cofactors was investigated for the engineered endonuclease, R.EcoR124I_NT_. The plasmids pUC19 (2686bp) containing two recognition sites at positions 1126 bp and 2294 bp and pTK-neo (2872 bp) containing one site starting at 2535 bp were used as the supercoiled DNA substrates. In addition, both plasmids were incubated with EcoRI to form linearised substrates with one and two sites, respectively (Fig. 1A).

In both assays, R.EcoR124I_NT_ was first formed by the addition of the R subunit to M.EcoR124I_NT_ that had previously been incubated at 37°C for 10 minutes in a buffer containing 10 mM Tris-HCl pH 8.0, 1 mM DTT, to give a final enzyme concentration of 200nM. The DNA substrate was then added to give a 20∶1 ratio of enzyme to DNA.

The reactions were started by the addition of 2 mM ATP. The assays were carried out in the absence and presence of 10 mM MgCl_2_ or AdoMet and incubated for 1 hour at 37°C (See Figure S1). In the absence of Mg^2+^ ions, no cleavage took place. ATP and magnesium ions were found to be absolutely required for cleavage of both supercoiled and linear DNA, whilst AdoMet was not required for cleavage (no significant effect on activity was seen up to 200µM).

The requirement for linear DNA to contain two recognition sites has been demonstrated for the wild-type enzyme EcoR124I, where the recognition sites can be in repeated or inverted orientation [20]. Cleavage of linear and circular DNA containing one or two recognition sites was investigated to determine which DNA substrate R.EcoR124I_NT_ cleaves more readily. Incubation with two-site supercoiled DNA (Fig. 1B) resulted in a discrete linear band via a nicked circle intermediate, as found for the native enzyme [19], [20]. Further investigation of the cleavage kinetics of the two-site supercoiled substrate showed that R.EcoR124I_NT_ rapidly cleaves the supercoiled DNA to a nicked circle intermediate, which is then linearised at a considerably slower rate (Figure 1D and 1E).

Incubation of R.EcoR124I_NT_ with a two-site linear DNA substrate yields a smear representing a range of DNA fragments (Fig. 1B). Densitometry of the gel shows that the smear covers a range of approximately 1 to 2 Kbp, with a peak at around 1.5 Kbp (Fig. 1C). This result suggests that cleavage occurs at sites roughly equidistant between the two recognition sites. The observed cleavage pattern is consistent with the mechanism whereby two type I enzymes cleave the DNA when they collide following translocation.

For linear DNA, two-site substrates are digested more rapidly than single-site substrate (for which no cleavage at all is detected in our assays (Fig. 1B)). However, for supercoiled DNA, the reaction proceeds at similar rates with either one- or two-site substrates, as also found for the native enzyme [19], [20]. It is more difficult to make quantitative comparisons of the absolutes rates, since we purified the R2 form of the enzyme and Janscak et al. [19] purified the R1 form. Moreover, the cleavage rates have been shown to vary non-linearly over a range of endonuclease concentrations and/or enzyme:substrate ratios [19], possibly reflecting partial dissociation of the subunits at sub-micromolar levels. We note that the enzyme concentrations usually used, both here and in previous studies of the native enzyme [19], [20], are at or below the K_d_ for binding of one of the R-subunits, which will strongly influence restriction activity (since both R subunits are required for cleavage).

### Subunit Assembly

Sedimentation velocity and electrophoretic mobility shift assays (EMSAs) were carried out in order to characterise the subunit assembly of the R.EcoR124I_NT_ restriction endonuclease and confirm the suitability of the complex for structural studies. For both sets of experiments, the methyltransferase (M.EcoR124I_NT_), the R1 complex (HsdR incubated with MTase at a 1∶1 molar ratio) and the restriction endonuclease, R.EcoR124I_NT_ (HsdR incubated with MTase at 2∶1 molar ratio) were then incubated with a 30 bp DNA duplex containing the recognition sequence for EcoR124I_NT_, each at a 1∶1 molar ratio of the enzyme: DNA.

**Figure 2:**
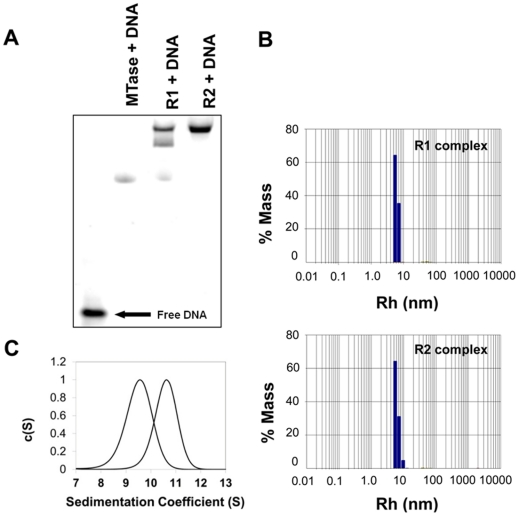
Assembly of R.EcoR124I_NT_. **(a)** Electrophoretic mobility shift assay (EMSA)**.** A 6% native gel was run at 150 V for 2.5 hours. Final concentrations of MTase and DNA were 2 µM. Lanes labelled R1 and R2 correspond to 1∶1 and 2∶1 molar ratios of HsdR to MTase. No difference was observed in the presence of 10 mM MgCl_2_. **(b)** Dynamic light scattering. The R1 and R2 complexes had hydrodynamic radii of 6.1 and 6.2 nm, respectively. **(c)** Sedimentation coefficient distributions of R.EcoR124I_NT_ (the R2 complex) plus and minus DNA. Sedimentation velocity data were collected at 285 nm, scanning every 12 minutes at 10°C at 30,000 rpm. Peak sedimentation coefficients for the free enzyme (9.5 S) and the enzyme bound to DNA (10.6 S) were converted to S_20,w_ values of 12.6 S and 14.0 S, respectively.

EMSAs revealed a full shift to the R2 complex at a 2∶1 ratio of R:MTase, while a mixture of R1 and R2 complex was observed at a 1∶1 ratio (Fig. 2A). In fact the latter result shows that the R2 complex predominates, even at a 1∶1 ratio, suggesting that there is cooperativity between the first and second R subunit binding events. This may explain, at least in part, the very different binding affinities for the two subunits [21]. The R1 complex of the wild-type ENase, has been shown to lack endonuclease activity and is able to translocate DNA at the same rate as the R2 complex, but with decreased processivity [22], [23]. Dynamic light scattering was also used to characterize the behavior of the complexes in solution, showing that the R1 and R2 complexes had hydrodynamic radii of 6.1 nm and 6.2 nm respectively (Fig. 2B).

Sedimentation velocity experiments were performed with free protein and with protein-DNA complexes, in both cases at 1∶1 and 2∶1 ratios of the R : MTase. The ENase (R2 complex) formed at a 2∶1 ratio showed single species with sedimentation coefficients (after correction to s^o^_20,w_) of 12.6 S and 14.0 S in the absence and presence of DNA respectively (Fig. 2C). In contrast, the sample formed at a 1∶1 ratio showed a mixture of species, including complexes with similar sedimentation coefficients to the above, as well as a mixture of free MTase and R, consistent with results from EMSAs (data not shown). We have previously shown the R subunit to be monomeric in solution at similar concentrations [24].

### Low-resolution Structure of the Multi-subunit Endonuclease

In order to determine the shape of R.EcoR124I_NT,_ small-angle neutron scattering (SANS) experiments were performed. Small-angle neutron scattering can provide information such as the molecular mass of the sample, the radius of gyration (R*_g_*), overall maximum length (D*_max_*) and allows a low-resolution model to be reconstructed from the data. In addition, with deuteration of specific subunits and contrast variation methods, the relative location of each subunit within the complex can be determined.

**Figure 3:**
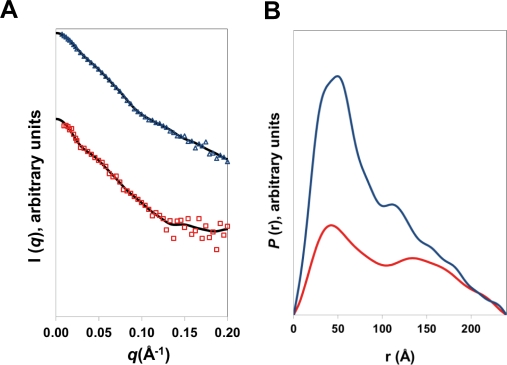
Small-angle neutron scattering of R.EcoR124I_NT_. **(a)** the SANS profile of R.EcoR124I_NT_ measured in H_2_O (blue triangles) and the SANS of the two HsdR subunits *in situ* from a sample containing deuterated HsdR subunits and protonated MTase, measured in 40% D_2_O (red squares). The solid black lines, show the fits from the back-transformed *P*(*r*) functions **(b)** Distance distribution function, *P*(*r*), obtained from the scattering profiles shown in (a).

The fully protonated native ENase was formed at a 2∶1 molar ratio of HsdR:MTase as described above and the concentrations used in the SANS experiments (ca. 3µM) are similar to those used in AUC and EMSA, where the ENase was shown to form an intact complex. The scattering data (Fig. 3) yielded a radius of gyration of 69 Å and D*_max_* of 240 ± 10 Å. These parameters are consistent with observations made for the wild-type ENases, R.EcoKI and R.EcoR124I.^12^ From analysis on an absolute scale [25], a molecular mass of 431 kDa was calculated for R.EcoR124I_NT_, in good agreement with the expected theoretical mass of the complex (405 kDa).

To obtain the position of each R subunit within the complex, small-angle neutron scattering experiments were performed in 40% D_2_O on the ENase that had been reconstituted with deuterated R subunits. Under these conditions, the shape and position of the HsdR subunits can be determined, as the scattering density of the methyltransferase “core” is matched out. From these measurements, we obtained an R*_g_* of 69 Å and D*_max_* of 240 Å. The calculated molecular mass of 230 kDa, was in good agreement with the theoretical value for two HsdR subunits (2×120 kDa).

**Figure 4:**
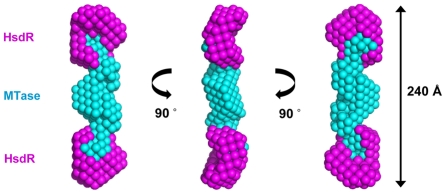
Multi-phase dummy atom modelling (MONSA) of R.EcoR124I_NT_. The phase representing the MTase is shown in cyan and the HsdR subunits are in magenta. The model was obtained by simultaneously fitting the SANS data shown in Figure 3. The three views are related by successive 90° rotations around the long axis of the complex.

Based on these parameters and the overall dimensions and shape of the methyltransferase, previously determined by SANS [11], it appears that the two R subunits lie at the outer extremes of the complex, either side of the M and S subunits of the MTase, with the S subunits centrally located. A more detailed model of R.EcoR124I_NT_ was then reconstructed by the multi-phase dummy atom modelling program MONSA [26], where the deuterated R-subunits can be assigned a different scattering density to the MTase subunits. The resulting shape of the ENase derived from the MONSA analysis has dimensions of around 240 × 90 × 55 Å. The methyltransferase component is located centrally within the envelope of the restriction endonuclease, while each HsdR subunit is located towards to outermost edge of the complex (Figure 4).

## Discussion

Our SANS results demonstrate that the subunit architecture and overall shape of the engineered, symmetrical endonuclease closely resembles the wild-type enzyme [12]. The two modified S_NT_ subunits, corresponding to just over half an intact S subunit, are able to dimerise effectively via the coiled-coil interface to form a structure that is homologous to the native S subunit containing two TRD’s, but with dyad symmetry. Clearly, covalent interactions between the two halves of the S subunit are not required for subunit assembly, for ATP-driven DNA translocation or for DNA cleavage.

The question then arises of why a more complex RM system has evolved, in which the two TRDs of the specificity subunit are fused together, since this is not a requirement for any aspect of enzyme activity. It is likely that the evolution of Type I RM systems with such fused TRD’s represents an evolutionary advantage in allowing asymmetrical DNA recognition sequences. For example, for a symmetrical hexanucleotide recognition site (defined by a unique trinucleotide sequence), the number of possible restriction sequences is 64 ( =  4^3^) base pairs. For an asymmetrical sequence with two independent trinucleotide sequences, there are 4096 ( =  4^6^) possible sequences (and the variation in the length of the spacer between half-sites found in Type I enzymes leads to a further increase in specificity). It is clear that the evolution of Type I RM systems in which the TRDs are fused into a single polypeptide greatly expands the possible repertoire of specificities available.

## Materials and Methods

### Protein Expression and Purification

M.EcoR124I_NT_ and HsdR from EcoR124I, were expressed and purified as described previously [11], [24]. Purified protein was subsequently dialysed into buffer A (10 mM Tris.HCl pH 8.0, 100 mM NaCl, 1 mM Na_2_EDTA).

### Preparation of Oligonucleotide Duplexes

Oligonucleotides (Invitrogen) corresponding to each strand of the EcoR124I_NT_ recognition sequence were mixed at equi-molar ratios and heated to a temperature of 90°C for 10 min in buffer A, supplemented with 10 mM MgCl_2_. The recognition sequence is shown in bold, while the non-specific spacer sequence is underlined.

5′- CCGTGCA**GAA** TCCGAGG **TTC**ACGGATCCGG –3′

5′- CCGGATCCGT**GAA** CCTCGGA **TTC**TGCACGG –3′

The molar extinction coefficient of the duplex was taken as E_260_  =  396,000 M^−1^ cm^−1^. A non-denaturing polyacrylamide gel at a 16% acrylamide concentration was used to confirm duplex formation.

### Formation of Protein and Protein-DNA Complexes

The R1 and R2 complexes of EcoR124I_NT_ were formed by mixing purified HsdR and M.EcoR124I_NT_ at 1∶1 and 2∶1 molar ratios, respectively. Protein-DNA complexes were formed by mixing M.EcoR124I_NT_, R1.EcoR124I_NT_ and R2.EcoR124I_NT_ with the DNA duplex at a 1∶1 molar ratio.

### Restriction Endonuclease Assay

The plasmids: pUC19 (Invitrogen, 2686bp) containing two recognition sites at positions 1126 bp and 2294 bp (GAACCCCCCGTTC and GAAAACGTTCTTC, respectively), and pTK-neo (Novagen, 2872 bp) containing one site at position 2535 bp (GAACGGGGGGTTC) were used as the DNA substrates. HiSpeed^TM^ Plasmid Maxi Kit (Qiagen) was used to purify the starting plasmid-DNA substrates. To form linearised substrates with one or two sites, the plasmids were digested with EcoRI.

The assays were carried out in NEB buffer 2 (50 mM NaCl, 10 mM Tris-HCl, 10 mM MgCl_2,_ 0.5 mM EDTA, 1 mM Dithiothreitol pH7.9). M.EcoR124I_NT_ and HsdR were mixed at a 1∶2 molar ratio (200 nM final enzyme concentration) with 10 nM DNA and incubated for 15 minutes at 37 °C. 2 mM ATP was added to start the reactions. During a time course experiments, 15 µL aliquots were removed at different times and inactivated by the addition of 5 µL 0.5M EDTA pH 8.0. The products of the reactions were run on a 0.8% agarose gel containing ethidium bromide at 0.5µg/ml, for 4 hours at 80 V. The gels were scanned with a phosphorimager (Fujifilm FLA−5000).

### Electrophoretic Mobility Shift Assay (EMSA)

Complexes were formed as described above. Final concentrations were 2 µM for MTase and DNA and 2 or 4 µM for HsdR (to form R1 and R2 complexes). Complexes were mixed with 5×Ficoll loading buffer (Buffer A plus 10 mM MgCl_2_ and 20% w/v Ficoll). Samples were loaded onto a 6% non-denaturing polyacrylamide gel and electrophoresis was carried out at 150 V for 2.5 hours in 0.25×TBE. The gel was stained with Ethidium Bromide and visualised on a phosphorimager (Fujifilm FLA-5000).

### Dynamic Light Scattering

Dynamic light scattering (DLS) was performed on purified R1 and R2 complexes at approximately 3µM, at 10°C in buffer A, using a Protein Solutions DynaPro MSTC800 light scattering instrument. The results from 30 measurements were averaged, and values for the hydrodynamic radius, R_h_, and polydispersity were obtained. The experimental molecular mass, M_r_, was estimated using the standard molecular weight model (Dynamics V5, Protein Solutions).

### Sedimentation Velocity

Sedimentation velocity experiments were carried out in a Beckman Optima XL-A analytical ultracentrifuge (Beckman-Coulter, Palo Alto, CA). 400 µL of sample and 425 µL of buffer A were loaded into the corresponding sectors of a double sector cell of 12 mm optical path length. The cells were loaded into an AN50-Ti analytical rotor, equilibrated overnight at 4°C. The rotor was accelerated to 30,000 rpm and readings of absorbance versus radial distance were taken every 12 minutes at 280 nm at 10°C. The raw data were analysed with the program SEDFIT [27] using radial data within the range 6.06 – 7.00 cm. Partial specific volumes and buffer densities were calculated with the program SEDNTERP and corrected for temperature [28]. The experimental sedimentation coefficients obtained from the c(s) distribution plot were finally corrected for temperature and buffer composition with SEDNTERP, to give s_20,w_ values for each component.

### Small-angle Neutron Scattering

HsdR (EcoR124I) was deuterated by first adapting BL21 (DE3) bacterial cells [11] containing the expression plasmid pBGSR124 encoding the hsdR gene. Enfors minimal medium, containing 85% D_2_O with hydrogenated glycerol as the carbon source, was used to give a 75% deuteration level, such that the protein had a contrast match point of 100% D_2_O [11]. Complexes of R.EcoR124I_NT_ were formed either as a fully hydrogenated enzyme or a perdeuterated complex. Complexes were then dialysed into buffer A containing varying H_2_O/D_2_O ratios. Polydispersity was monitored by dynamic light scattering, prior to SANS analysis, and confirmed the lack of aggregated species.

Data were collected using the D22 diffractometer at the ILL using two detector distances at 2m and 7m, for 15 and 30 minutes, respectively, with a wavelength of 6 Å, covering a *q* range of 0.007 to 0.35 Å^−1^, where q is the scattering vector (4πsinθ/λ). Scattering data was collected from a 96 × 96 cm detector with a pixel size of 7.5 × 7.5 mm. Data reduction was performed using the GRASansP software. Modelling of the SANS data was performed using the ATSAS software package (Version 2.4). The final merged scattering data covered the q range 0.007 to 0.20 Å^−1^, and was further evaluated using PRIMUS [29]. At low angle, the isotropic scattering data can be expressed as the Guinier approximation, I(q)  =  *I*(0) exp 1/3 R*_g_* ^2^q^2^ [30]. The isotropic scattering intensity, I(q) was transformed to the distance distribution function *P*(*r*) using the program GNOM [31], which was also used to calculate the particle maximum dimensions D*_max_*. Fits to the experimental scattering curve were generated by back transformation of the *P*(*r*) function. The optimum value of D*_max_* was found when the R*_g_* obtained from the *P*(*r*) plot was equal that obtained from the Guinier analysis.

### *Ab Initio* Modeling

*Ab initio* modelling of the ENase_NT_ was performed with the multi-phase dummy-atom modelling program MONSA [26]. This program attempts to minimise the discrepancy between the fit of the model and the experimental data, describing the model by an assembly of beads within a spherical search volume with a diameter equal to that of the D_max_ of the complex. The model was iteratively fitted to two SANS datasets, the 0% D_2_O SANS data containing information on the overall shape of the complex and the 40% D_2_O SANS data that describes the location of the deuterated HsdR subunits. Two phases were specified, one for HsdR subunits and the other, for the MTase component. The latter component had been already derived by SANS [11], and was used as part of the dummy atom model (DAM) input into MONSA. The resulting file was edited such that the MTase phase would be maintained during the modelling process. This approach assumes no significant change in structure of the MTase “core” when the HsdR binds, as also suggested from observations on the wild-type MTase [12].

The MTase model was moved to the origin with MASSHA [32] and a spherical search volume with a diameter of 240Å was created using the auxiliary ATSAS program “pdb2dam4” (kind gift from Maxim Petoukhov). The following edits were made to the pdb output to maintain the MTase phase during MONSA: "H_space_” was replaced with “CA”, “0 1 201” was replaced with “0 1 202” and “0 2 201” was replaced with “1 2 201”. Theoretical volumes based on the amino acid sequence and R_g_ values determined from Guinier analysis were used as further constraints during the modelling process, as was the overall R_g_ of the complex. P2 symmetry was also imposed. The program MULCh: ModULes for the analysis of contrast variation data [33] was used to calculate the theoretical value of the scattering length densities (and therefore the contrast) for each H_2_O/D_2_O level, based on the amino acid sequence for the protein and buffer composition. All models were visualized using the program PYMOL [34]. 

## Supporting Information

Figure S1 **DNA cleavage in the presence and absence of cofactors.** The endonuclease was incubated with puC119 (3127bp), a supercoiled plasmid containing two recognition sites at positions 1168 and 1293, in the absence (**A**) and in the presence (**B**) of 10 mM MgCl2. Lane M represents a KiloBaseTM DNA marker (GE Healthcare). Lanes SC and L, represent supercoiled and linear DNA controls. Lane 1 represents the reaction at 0 minutes and lanes 2 and 3, represent the reactions in the absence or presence of 200 µM AdoMet, respectively, after a 60 minute incubation at 37°C.
(TIFF)
