# Correction: Structural and Functional Analysis of the Symmetrical Type I Restriction Endonuclease R.EcoR124I_NT_

**DOI:** 10.1371/journal.pone.0230278

**Published:** 2020-03-04

**Authors:** James E. N. Taylor, Anna Swiderska, Jean-Baptiste Artero, Philip Callow, Geoff Kneale

The following information is missing from the Funding statement: This study was also supported by the Engineering and Physical Sciences Research Council (grants GR/R99393/01 and EP/C015452/1) to GK.
