# Correction: Structural and Functional Analysis of the Symmetrical Type I Restriction Endonuclease R.EcoR124I_NT_

**DOI:** 10.1371/journal.pone.0301486

**Published:** 2024-03-26

**Authors:** James E. N. Taylor, Anna Swiderska, Jean-Baptiste Artero, Philip Callow, Geoff Kneale

The first author’s name is incorrectly listed without the middle initial ’N’. The correct name is: James E. N. Taylor. The correct citation is: Taylor JEN, Swiderska A, Artero J-B, Callow P, Kneale G (2012) Structural and Functional Analysis of the Symmetrical Type I Restriction Endonuclease R.EcoR124I_NT_. PLoS ONE 7(4): e35263. https://doi.org/10.1371/journal.pone.0035263.
