# Supplementary figures and images for: Structural and Functional Analysis of the Symmetrical Type I Restriction Endonuclease R.EcoR124INT
Source: PLoS One. 2012 Apr 6;7(4):e35263. doi: 10.1371/journal.pone.0035263 (PMC3320862; doi:10.1371/journal.pone.0035263)

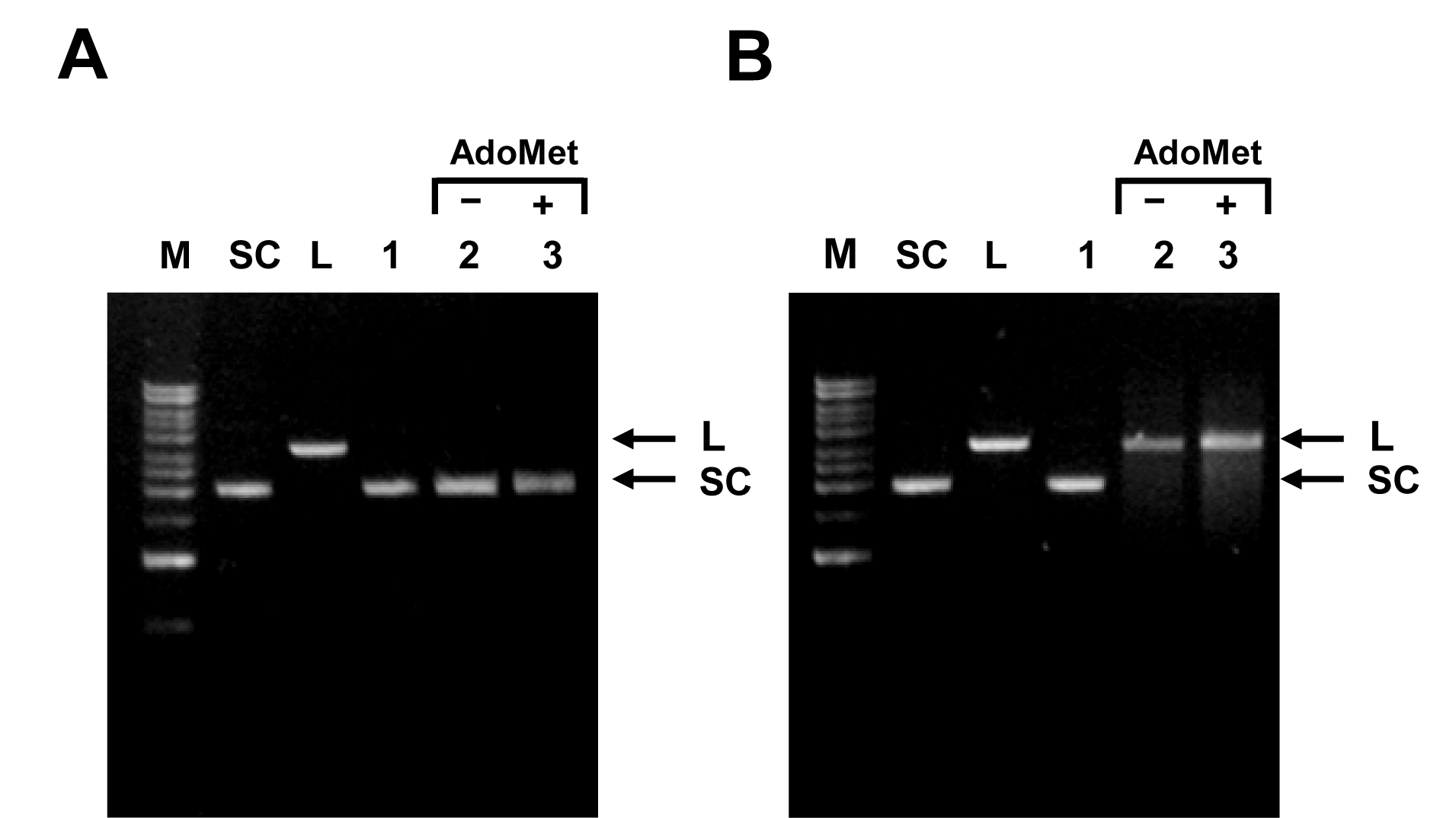

Supplement: Figure S1 — DNA cleavage in the presence and absence of cofactors. The endonuclease was incubated with puC119 (3127bp), a supercoiled plasmid containing two recognition sites at positions 1168 and 1293, in the absence (A) and in the presence (B) of 10 mM MgCl2. Lane M represents a KiloBaseTM DNA marker (GE Healthcare). Lanes SC and L, represent supercoiled and linear DNA controls. Lane 1 represents the reaction at 0 minutes and lanes 2 and 3, represent the reactions in the absence or presence of 200 µM AdoMet, respectively, after a 60 minute incubation at 37°C. (TIFF) [file pone.0035263.s001.tiff]
